# Dynamic Roles for Small RNAs and DNA Methylation during Ovule and Fiber Development in Allotetraploid Cotton

**DOI:** 10.1371/journal.pgen.1005724

**Published:** 2015-12-28

**Authors:** Qingxin Song, Xueying Guan, Z. Jeffrey Chen

**Affiliations:** 1 Department of Molecular Biosciences, Institute for Cellular and Molecular Biology, Center for Computational Biology and Bioinformatics, The University of Texas at Austin, Austin, Texas, United States of America; 2 State Key Laboratory of Crop Genetics and Germplasm Enhancement, Nanjing Agricultural University, Nanjing, China; Harvard Medical School, UNITED STATES

## Abstract

DNA methylation is essential for plant and animal development. In plants, methylation occurs at CG, CHG, and CHH (H = A, C or T) sites via distinct pathways. Cotton is an allotetraploid consisting of two progenitor genomes. Each cotton fiber is a rapidly-elongating cell derived from the ovule epidermis, but the molecular basis for this developmental transition is unknown. Here we analyzed methylome, transcriptome, and small RNAome and revealed distinct changes in CHH methylation during ovule and fiber development. In ovules, CHH hypermethylation in promoters correlated positively with siRNAs, inducing RNA-dependent DNA methylation (RdDM), and up-regulation of ovule-preferred genes. In fibers, the ovule-derived cells generated additional heterochromatic CHH hypermethylation independent of RdDM, which repressed transposable elements (TEs) and nearby genes including fiber-related genes. Furthermore, CHG and CHH methylation in genic regions contributed to homoeolog expression bias in ovules and fibers. Inhibiting DNA methylation using 5-aza-2'-deoxycytidine in cultured ovules has reduced fiber cell number and length, suggesting a potential role for DNA methylation in fiber development. Thus, RdDM-dependent methylation in promoters and RdDM-independent methylation in TEs and nearby genes could act as a double-lock feedback mechanism to mediate gene and TE expression, potentiating the transition from epidermal to fiber cells during ovule and seed development.

## Author Summary

Cotton is the world’s largest source of renewable textile fiber and is an allotetraploid crop consisting of two progenitor genomes. In plants, *de novo* CHH (H = A, T, or C) methylation depends on RNA-directed DNA methylation (RdDM) and CHROMOMETHYLASE2 (CMT2)-mediated pathways. The biological significance of the two pathways is largely unknown. Here we show dynamic roles of these two pathways in ovule and fiber development. RdDM-dependent CHH methylation is linked to gene activation in ovules, and additional CMT2-dependent methylation leads to silencing of transposons and nearby genes in fibers. Moreover, DNA methylation affects expression bias of homoeologous genes and fiber development. These findings provide novel insights into epigenetic regulation of organ development and polyploid evolution.

## Introduction

DNA methylation, a conserved epigenetic mark in most eukaryotes, is essential for growth and development and is associated with many epigenetic phenomena, including imprinting and transposon silencing [1–5]. In plants, DNA is methylated in CG, CHG and CHH (H = A, T, or C) sites through distinct pathways. In *Arabidopsis*, CG methylation is maintained by METHYLTRANSFERASE1 (MET1), a homolog of mammalian DNMT1 [6]. Plant-specific CHROMOMETHYLASE3 (CMT3) is primarily responsible for CHG methylation, which is coupled with H3K9 dimethylation [7]. CHH methylation is established *de novo* by DOMAINS REARRANGED METHYLTRANSFERASE1 and 2 (DRM1 and DRM2) [8] through the RNA-directed DNA methylation (RdDM) pathway [9], involving 24-nt small interfering RNAs (siRNAs) [1, 2]. Recent studies found that CHH methylation could also be established by CMT2 [10, 11], through histone H1 and DECREASE-IN-DNA-METHYLATION1 (DDM1) activities [12], which is independent of the RdDM. The methylome data indicate that CMT2 and RdDM pathways preferentially function in heterochromatic and euchromatic regions, respectively [10, 11]. However, the role for DNA methylation in developmental regulation is poorly understood.

Cotton is the largest source of renewable textile fiber and an excellent model for studying the developmental transition from ovule epidermal cells to rapidly-elongating singular fiber cells. The most widely-cultivated cotton (*Gossypium hirsutum* L., AADD) is an allotetraploid species, which originated 1–2 million years ago from interspecific hybridization between A-genome species, resembling *Gossypium herbaceum* or *Gossypium arboretum*, and D-genome species, resembling *Gossypium raimondii* [13]. The intergenomic interaction in allotetraploid cottons induces longer fiber and higher yield, coincident with expression bias of fiber-related homoeologous genes [14, 15], which provides the basis of selection and domestication for agronomic traits in cotton and many other polyploid crops [13, 16]. Each cotton fiber is a single cell derived from the ovule epidermis, undergoing rapid cell elongation and cellulose biosynthesis, and ~100,000 fiber cells develop semi-synchronically in each ovule (seed) and can reach six centimeters in length [17, 18]. In early stages of fiber development, rapid cell growth is associated with a dramatic increase of DNA content by endoreduplication [19, 20] and dynamic changes in gene expression and small RNAs [15, 18, 21]. Interestingly, DNA methylation changes are related to seasonal variation of fiber development in cotton [22] and is also shown to change among different tissues including fibers based on the methylation-sensitive high-performance liquid chromatography (HPLC) assay [23]. Moreover, over-expressing fiber-related transgenes often leads to the unexpected outcome of fiber phenotypes [24]. These data indicate a potential role for DNA methylation in gene expression and phenotypic traits such as cotton fiber, which could be selected and domesticated.

Although genome-wide DNA methylation has been examined in *Arabidopsis* [25], soybean [26, 27], maize [28, 29], and other plants and animals [30, 31], the roles of RdDM and CMT2-dependent methylation pathways in organogenesis and development remain elusive. In this study, we employed cotton ovule and fiber cells as a model to test the role of DNA methylation in developmental regulation. Using methylcytosine-sequencing (MethylC-seq) [32, 33], RNA-seq, and small RNA-seq analyses, we examined CG, CHG, and CHH methylation patterns in fibers, ovules, and leaves and analyzed differentially methylated regions (DMRs) between the ovule and leaf (OL) and between the fiber and ovule (FO). The methylation patterns in the gene body and 5’ and 3’ flanking sequences were comparatively analyzed with TE densities and expression levels of genes and small RNA loci. The results support unique roles of CG, CHG, and CHH methylation in ovule and fiber development and expression bias of homoeologous genes in the allotetraploid cotton.

## Results

### DNA methylation dynamics in cotton leaves, ovules, and fibers

**Fig 1:**
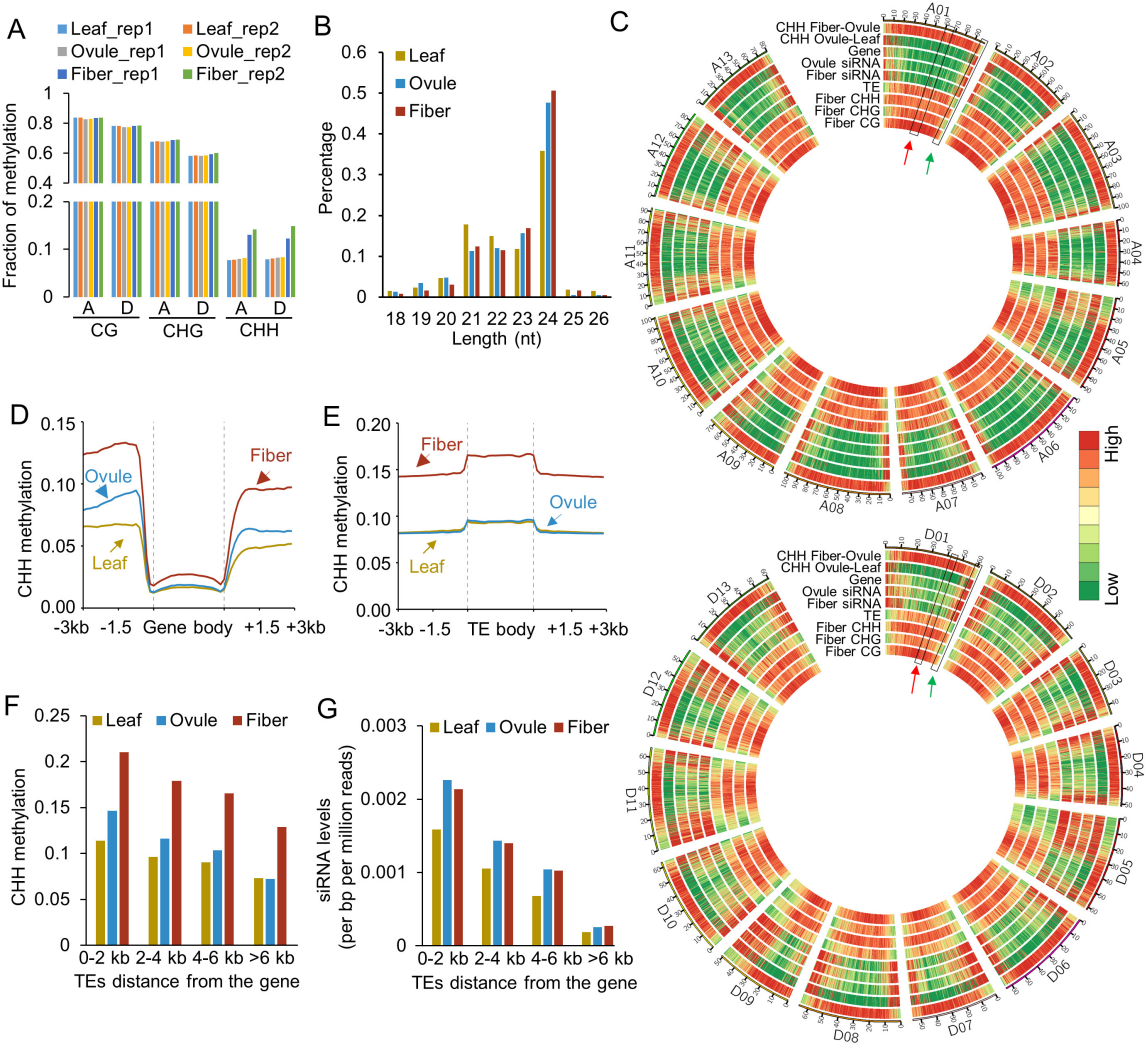
Effects of DNA methylation on ovule and fiber development and genome-wide distribution of DNA methylation in leaves, ovules, and fibers. (A) Percentage of methylated cytosine (mC) in leaves, ovules (0 DPA), and fibers (14 DPA). A and D indicate A- and D subgenomes in the allotetraploid cotton, respectively. (B) Size distribution of small RNAs in different tissues. (C) Circle plots of fiber CG, CHG, CHH methylation, fiber small RNA density, gene density, TE density, and ovule-leaf and fiber-ovule CHH methylation using 100-kb windows among 13 A-homoeologous chromosomes (upper) and 13 D-homoeologous chromosomes (lower). Color bars indicate low (green) to high (red) densities or intensities. The scales are: fiber CG and CHG methylation level: 0–1; fiber CHH methylation level: 0–0.18; TE density: 0–70%; Gene density: 0–20%; siRNA density in fiber and ovule: 0–60 reads per million reads; Ovule-fiber CHH methylation: 0–0.01; fiber-ovule CHH methylation: 0–0.07. (D) Distribution of CHH methylation in genes. (E) Distribution of CHH methylation in TEs. (F) CHH methylation levels in TEs relative to the distance from the nearest gene. (G) siRNA levels (per bp per million reads) in TEs relative to the distance from the nearest gene.

DNA methylation affects growth and development in plants and animals [3, 4]. To investigate genome-wide DNA methylation changes during ovule and fiber development, we used allotetraploid cotton (*G*. *hirsutum* L. acc. TM-1) to perform whole-genome bisulfite sequencing in leaves, ovules at 0 DPA, and fibers at 14 DPA with two biological replicates and ovules at 14 DPA with one replicate. The TM-1 sequence of A and D subgenomes [34] was used as the reference for data analysis. The bisulfite-conversion rates were over 99% (S1 Table). Approximately 80% of cytosines were covered by at least one uniquely mapped read, and the mean coverage of cytosines was 8.2-fold or higher for all tissues. Methylation levels between biological replicates were highly correlated among symmetric sites (Pearson *r*>0.9) and all sites (*r*>0.8), indicating reproducibility of the data (Figs 1A and S1A). Overall, CG and CHG methylation levels were similar among fibers, ovules and leaves but were lower in the D subgenome than in the A subgenome for all tissues (Fig 1A). However, CHH methylation levels were much higher in fibers (~14%) than in ovules (~8.1%) and leaves (~7.8%) (Fig 1A). This high methylation level in fibers was not related to the late developmental stage because the methylation level in ovules was slightly lower at 14 DPA than at 0 DPA (S1B Fig **)**. CHH methylation is induced by small RNAs through RdDM [2, 35]. To test a link of DNA methylation changes in cotton tissues with small RNAs, we generated small RNA-seq data with three replicates in leaves, ovules at 0 DPA, and fibers at 14 DPA (S1 Table). The data showed 24-nt siRNAs were much higher in fibers and ovules than in leaves (Fig 1B) [15].

On each chromosome, DNA methylation was more abundant in transposable element (TE)-rich than gene-rich regions in all tissues (Figs 1C and S1C). However, 24-nt siRNAs were highly enriched in gene-rich regions (Figs 1C and S1C), suggesting a role for these siRNAs in genic methylation. Because each fiber cell is expanded from an epidermal cell of the ovule, further comparison was made between the ovule and leaf (ovule-leaf, OL) and between the fiber and ovule (fiber-ovule, FO) methylation levels, which represented methylation changes in the ovule and fiber, respectively. OL CHH hypermethylation correlated with gene-rich regions, showing the same trend as that of the siRNAs (Fig 1C). On the contrary, FO CHH hypermethylation was enriched in TE- and repeat-rich regions, characteristic of heterochromatin. The data indicate that OL CHH hypermethylation preferentially occurs in euchromatic regions, whereas FO CHH hypermethylation predominates in heterochromatic regions (Figs 1C and S1D).

CHH methylation differences between the ovule and leaf occurred mainly in 5’ upstream and 3’ downstream sequences of the genes (Fig 1D), while CG and CHG methylation levels in genes and TEs were similar among different tissues tested (S2 Fig). Among all TEs, mean CHH methylation levels were higher in the fiber but indistinguishable between the ovule and leaf (Fig 1E). The higher CHH methylation levels of TEs in fibers and ovules were correlated with the closer distances of TEs to the gene (Fig 1F). When TEs were more than 4-6-kb away from the gene, CHH methylation levels became similar between the ovule and leaf. Consistently, the CHH methylation levels mirrored the 24-nt siRNA distribution patterns, which were high near the genes and decreased as they were further away from the gene, indicating the role of the RdDM pathway in CHH methylation of the gene nearby TEs (Fig 1G).

### Differentially methylated regions between the ovule and leaf and the fiber and ovule

**Fig 2:**
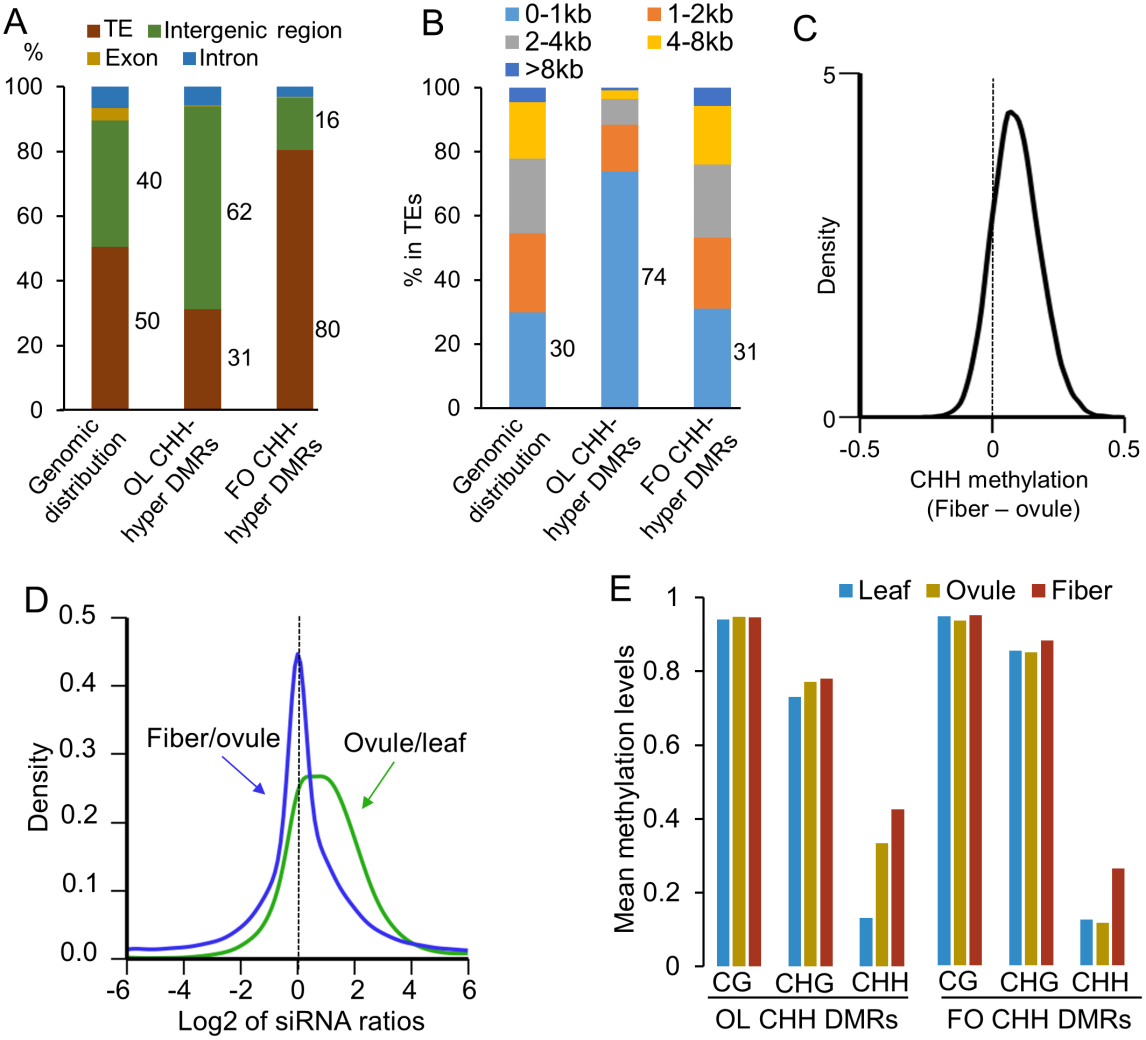
Genomic distribution of differentially methylated regions (DMRs). (A) Percentage of DMRs in TE (brown), intergenic region (green), exon (dark yellow) and intron (blue). (B) Percentage of DMRs corresponding to TEs with different sizes. (C) Kernel density plot of CHH methylation change of fiber/ovule in OL CHH-hyper DMRs. (D) Kernel density plot showing 24-nt siRNA fold-changes of ovule/leaf in OL CHH-hyper DMRs (green) and of fiver/ovule in FO CHH-hyper DMRs (blue). (E) Percentage of CG, CHG and CHH methylation in the leaf (blue), ovule (yellow), and fiber (red) in OL CHH-hyper DMRs and FO CHH-hyper DMRs.

As CG and CHG methylation levels were similar among different tissues examined, further analysis was focused on the CHH methylation changes among these tissues. We predict that differentially methylated regions (DMRs) between tissues play a role in biological function. To test this, we identified 39,668 CHH-hypermethylated DMRs in the ovule relative to leaf (ovule-leaf) (OL CHH-hyper DMRs) and 124,681 CHH-hypermethylated DMRs in the fiber relative to ovule (fiber-ovule) (FO CHH-hyper DMRs) (S2 Table **)**. A subset of these DMRs was validated by bisulfite-sequencing individually cloned genomic fragments (S3 Fig), confirming the results of these DMRs based on the genome-wide analysis. Compared to distributions of exons and introns in genomic distribution, both OL and FO CHH-hyper DMRs were underrepresented in exons and introns (Z-score <-5) (Fig 2A). However, OL CHH-hyper DMRs were overrepresented in intergenic regions (62%) relative to the average genomic distribution (40%), whereas FO CHH-hyper DMRs were enriched in TEs (80% vs. 50%). Among TEs, short TEs (less than 1-kb) were more abundant in OL CHH-hyper DMRs (74%) than in the genomic distribution (30%), whereas the percentage of short TEs in FO CHH-hyper DMR distribution was similar to the genomic distribution (Fig 2B). This suggests that OL hyper-CHH regions were located nearby genes (between genes with short TEs), whereas FO hyper-CHH regions are associated with TEs (genome-wide phenomenon).

Methylation levels of most OL CHH-hyper DMRs were higher in fibers than in ovules, indicating that OL CHH-hyper DMRs continue to be hypermethylated in elongating fibers after they developed from ovule epidermal cells (*P* < 1e^-200^, Wilcoxon rank-sum test) (Fig 2C). Interestingly, among the OL CHH-hyper DMRs, siRNA expression levels were significantly higher in ovules than in leaves (*P* < 1e^-100^, Wilcoxon rank-sum test), and this difference was not obvious between the fiber and ovule in FO CHH-hyper DMRs (Fig 2D). These data suggest that siRNAs induce CHH hypermethylation in the ovule, whereas the CHH methylation increase in the fiber was independent of the RdDM pathway. In addition to CHH methylation, OL CHH-hyper DMRs had slightly higher CG and CHG methylation levels in ovules (CG: 0.95; CHG: 0.77) than in leaves (CG: 0.94, CHG: 0.73), FO CHH-hyper DMRs also had higher CG and CHG methylation levels in fibers (CG: 0.96; CHG: 0.89) than in ovules (CG: 0.94; CHG: 0.85) (Fig 2E).

### Effects of OL CHH-hyper DMRs on gene expression

**Fig 3:**
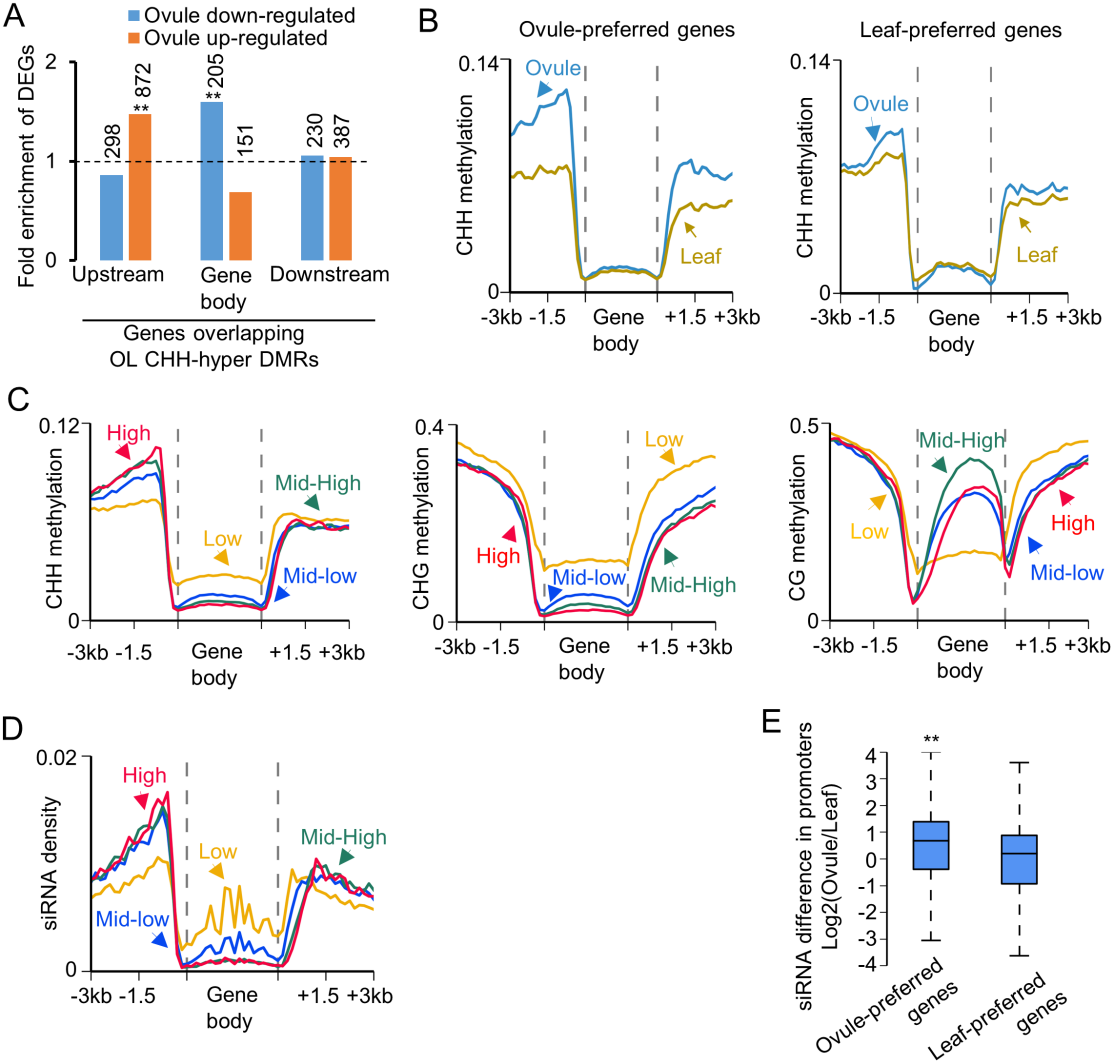
Relationship of ovule-leaf (OL) CHH-hyper DMRs and gene expression changes between the ovule and leaf. (A) Fold-enrichment of differentially expressed genes (DEGs) in those overlapping with OL CHH-hyper DMRs in flanking sequences or gene body, relative to all genes. The hypergeometric test was used to infer statistical significance (*: P < 0.05; **: P < 1e-10). Numbers above columns indicate upregulated genes (orange) and down-regulated genes (blue), respectively, in the ovule, which overlapped OL CHH-hyper DMRs. (B) CHH methylation distribution in genes of ovule-preferred and leaf-preferred genes in the leaf (yellow) and ovule (blue). Leaf-preferred and ovule-preferred genes were identified by both fold-change of gene expression (> 5-fold) and ANOVA test (< 0.01). (C) Relationships between CHH (left), CHG (middle) and CG (right) methylation and expression levels of genes in ovules, which were divided into four quartiles (high, red; mid-high, green; mid-low, blue; and low, yellow). (D) The relationship between siRNA density and the genes in the four quartiles as in (C). (E) Box plot showing siRNA abundance in promoter regions of ovule-preferred (left) and leaf-preferred (right) genes.

To test the role of methylation changes in gene expression, we compared RNA-seq data with DMRs between the ovule and leaf and/or between the fiber and ovule (S1 Table). Down-regulated genes in the ovule relative to the leaf were significantly enriched in the genes overlapping with OL CHH-hyper DMRs in the gene body (Fig 3A and S3 Table), which is consistent with the notion that CHH methylation in the gene body correlates negatively with gene expression [27, 36]. However, up-regulated genes in the ovule were significantly enriched in the genes overlapped with OL CHH-hyper DMRs in the upstream sequences (1 kb) (Fig 3A and S3 Table), indicating a positive correlation of CHH methylation in the upstream sequences with gene expression. For the genes that were preferentially expressed in the ovule (ovule-preferred genes), the CHH methylation level in the flanking sequences was higher in the ovule than in the leaf, whereas CHH methylation in flanking sequences of the leaf-preferred genes was similar between the ovule and leaf (Fig 3B). To further test the relationship between CHH methylation and gene expression, we divided genes into quartiles based on their expression levels in each tissue (from low to high) and compared them with DNA methylation changes. CHH and CHG methylation levels in the gene body were negatively associated with gene expression levels, and moderately transcribed genes showed the highest CG methylation level in the gene body (Figs 3C and S4), consistent with previous findings [27, 36]. Moreover, CHH methylation in the 5’ upstream coincided with siRNA abundance, which was positively associated with gene expression (Fig 3C and 3D). The siRNA levels in the promoter regions of ovule-preferred genes were significantly higher in the ovule than in the leaf (*P* < 1e^-4^, Wilcoxon rank-sum test) (Fig 3E). This positive correlation between CHH methylation and gene expression was unexpected but was also reported in other plants including maize [28], soybean [26], and rice [37]. Active transcription could induce hypermethylation, as shown in rice [37]. Alternatively, hypermethylation may induce gene expression.

**Fig 4:**
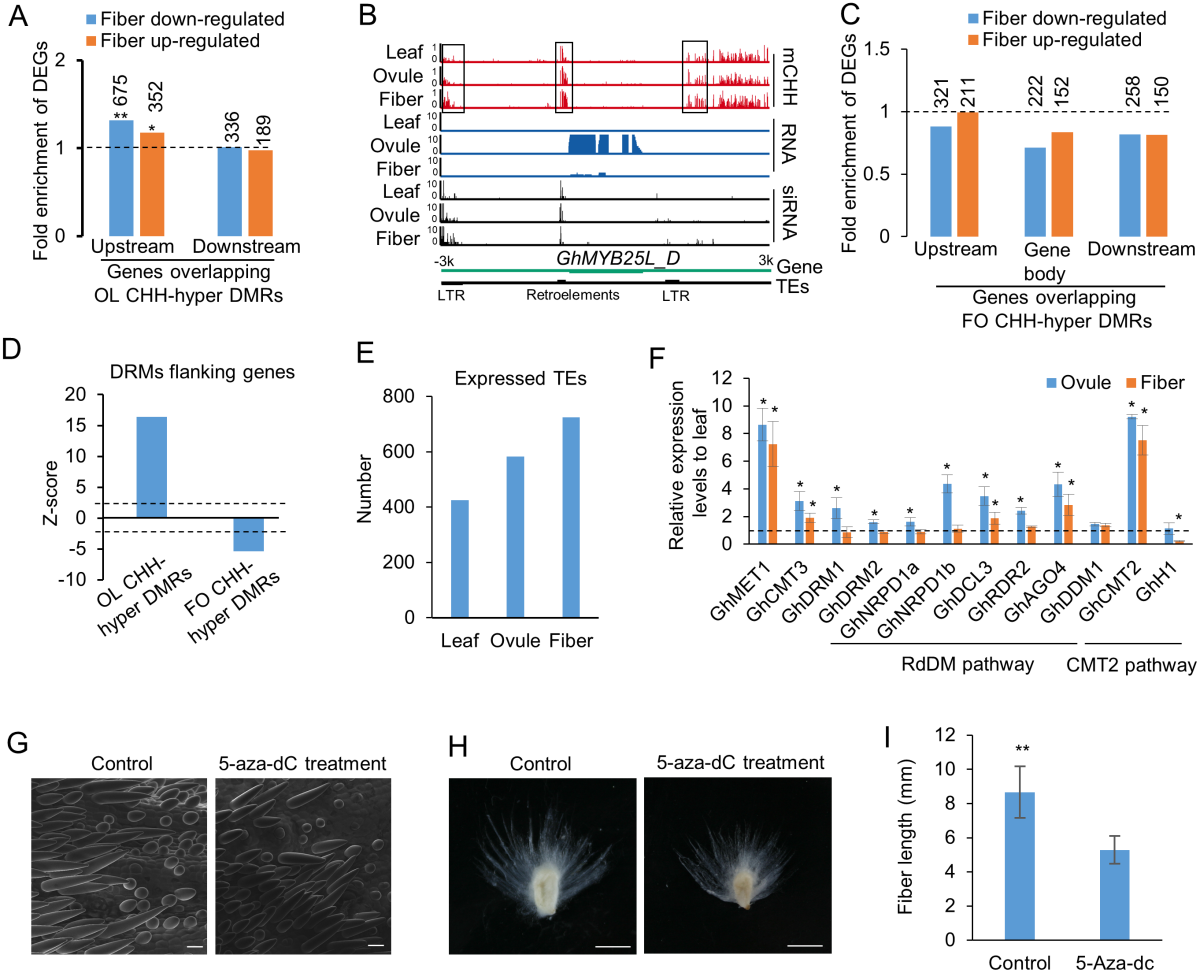
Influence of fiber-ovule (FO) CHH-hyper DMRs to gene expression and TE activity. (A) Fold-enrichment of DEGs between the fiber and ovule in the genes overlapping with OL CHH-hyper DMRs, which were further hypermethylated in fibers, relative to all genes. The hypergeometric test was used to infer statistical significance (*: P < 0.05; **: P < 1e-10). Numbers above columns indicate DEGs between fiber and ovule, which overlapped OL CHH-hyper DMRs. (B) An example of fiber CHH hypermethylation correlating with gene repression in the fiber. (C) fold-enrichment of DEGs between the fiber and ovule in the genes overlapping with FO CHH-hyper DMR in the gene body and flanking sequences, relative to all genes. Numbers above columns indicate DEGs between fiber and ovule in genes, which overlapped FO CHH-hyper DMRs. (D) Enrichment (z-score) of DMRs in flanking sequences (1 kb) of genes. The dash line indicates the statistical significant level (P < 0.05). (E) Number of expressed TEs in the leaf, ovule and fiber. (F) Relative expression levels of genes related to DNA methylation in the leaf, ovule and fiber. Cotton *HISTONE H3* was used as internal control for qRT-PCR analysis. The dash line indicates the expression level that is normalized to the leaf. ANOVA was used to infer statistical significance (*, P < 0.05). (G) Scanning electron micrograph (SEM) showing shorter and fewer fibers in ovules treated by 5-aza-dC. Ovules at -3—4 DPA were cultured in vitro for 4 days without (control) and with 5-aza-dC (10mg/L). (H) Shorter fibers in ovules after the aza-dC treatment for 14 days. (I) Quantitative analysis of fiber length in (H) (student’s test, p<0.01).

The higher methylation levels of most OL CHH-hyper DMRs in fibers than in ovules may suggest a role for increased methylation in the gene expression of fibers. To test this, we extracted 26,245 OL CHH-hyper DMRs that showed higher methylation levels (cut-off value >0.05) in fibers than in ovules to examine effects of these DMRs on gene expression in fibers. In contrast to the up-regulated genes in the ovule, down-regulated genes (352) in the fiber relative to the ovule were significantly (Hypergeometric test, P < 0.05) enriched in those that overlapped with the OL CHH-hyper DMRs in the upstream sequences (Fig 4A), suggesting that further increasing methylation levels of some OL CHH-hyper DMRs in the fiber could repress nearby genes. For example, expression of *GhMYB25L_D* (*Gh_D12G1628*), a key player for fiber development [24], was induced at the fiber initiation stage (0 DPA) but repressed in the elongation stage (Fig 4B). *GhMyb25L_D* is flanked by three short TEs corresponding to CHH methylation (boxed). The methylation levels in the flanking TEs of *GhMyb25L_D* increased in ovules at 0 DPA and continued to increase in fibers at 14 DPA. These data indicate that additional CHH methylation in elongating fibers may be correlated with inhibition of *GhMYB25L_D* expression during fiber development (Fig 4B).

### Function of FO CHH hypermethylation

To our surprise, FO CHH-hyper DMRs were not significantly correlated with expression changes in the fiber (Fig 4C and S4 Table). This was partly because FO CHH-hyper DMRs tended to be excluded from the gene body (Fig 2A) and from the flanking sequences (1-kb) of the gene (Fig 4D). Instead, FO CHH-hyper DMRs were enriched in TEs (Fig 2A), and FO CHH hypermethylation correlated positively with the TE density (S1D Fig). In plants, TE transcripts are generally repressed through DNA methylation. We examined whether CHH hypermethylation in heterchromatin in fiber inhibited TEs. Indeed, RNA-seq data showed that more TEs were expressed in fibers than in ovules or leaves (Fig 4E). These data suggest that endoreduplication in early fiber cells may loosen chromatin structure, leading to activation of TEs. More transcription could induce hypermethylation. As a result, CHH hypermethylation in fibers may serve as a feedback mechanism to repress TEs. Although both ovule and fiber had more 24-nt siRNAs than the leaf, only fiber showed the CHH methylation increase in heterochromatin compared to the leaf, suggesting that higher heterochromatic CHH methylation in the fiber is partially related to siRNA abundance.

This CHH methylation increase in the heterochromatin could be catalyzed by CMT2 through a DDM1-mediated process [10, 11]. To test this, we examined expression of *GhCMT2* (*Gh_D08G1665*, *Gh_A08G1371*), *GhDDM1* (*Gh_A12G0603*, *Gh_D12G2782*) and other genes related to DNA methylation in leaves, ovules and fibers. Compared with leaves, *GhCMT2*, *GhCMT3* (*Gh_A07G0385*, *Gh_D07G0449*) and *GhMET1* (*Gh_A05G3224*, *Gh_D04G0381*) were upregulated in ovules and fibers, and the genes involved in the RdDM pathway, including *DRM1* (*Gh_A09G0264*, *Gh_D04G0527*), *DMR2* (*Gh_D09G0266*, *Gh_A05G3114*), *RDR2* (*Gh_D12G2624*, *Gh_A12G2496*), *NRPD1* (*Gh_A08G1605*, *Gh_D08G1916*), and *NRPE1* (*Gh_A06G1549*, *Gh_D06G1919*), with an exception of *AGO4* (*Gh_A08G1752*, *Gh_D08G2707*) and *DLC3* (*Gh_D06G0845*), were up-regulated in ovules but not in fibers (Fig 4F). However, *HISTONE H1* (*Gh_D08G0660*, *Gh_A08G0565*), which is shown to impede the access of DNA methyltransferases to the heterochromatin [10, 38], was repressed in fibers but not in ovules (Fig 4F). These data suggest that up-regulation of *GhCMT2* and repression of cotton *HISTONE H1* in fibers may induce CHH hypermethylation in TEs and heterochromatin in fibers. To investigate the biological role of DNA methylation in fiber development, we harvested ovules at -3 to 0 days post anthesis (DPA) from the allotetraploid cotton *Gossypium hirsutum* L. acc. TM-1 and cultured them *in vitro* with or without treatments of the DNA methyltransferase inhibitor, 5-aza-deoxycytidine (5-aza-dC) [39]. Consistent with the hypermethylation observed in ovules and fibers, the 5-aza-dC treatment suppressed fiber length and ovule size in the initiation and elongation stages (Fig 4G, 4H and 4I). The data suggest that DNA methylation might be important for normal fiber and ovule development, although we cannot rule out possible side effects of growth inhibition by 5-aza-dC.

### Role of DNA methylation in the expression of homoeologous genes

**Fig 5:**
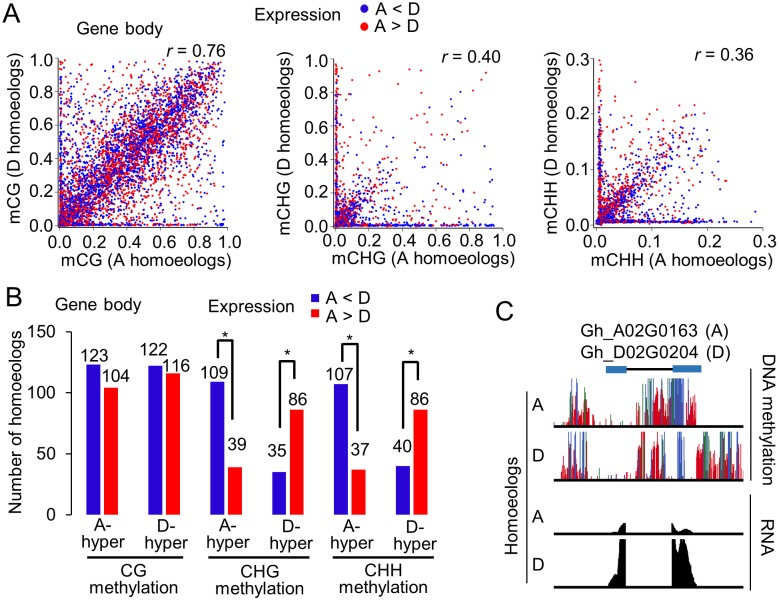
DNA methylation differences in homoeologous genes. (A) Pairwise plots of CG (left), CHG (middle), and CHH (right) methylation levels (Y-axis, D homologs; X-axis, A homoeologs) in the gene body for homoeologous genes. Blue and red dots indicate the expression level of an A homoeolog that is lower and higher than that of the corresponding D homoeolog, respectively. Spearman correlation coefficient (r) is shown. Fold-changes of expression (>2-fold) between A and D homoeologs were used as cut-off values. (B) Number of homoeologous genes that were differentially methylated in the gene body and showed expression differences (A < D, blue; A > D, red). The levels of CG (>0.4), CHG (>0.4), and CHH (>0.1) methylation difference and fold-changes of expression (>2-fold) between A and D homoeologs were used as cut-off values. (C) An example of hypermethylation in the gene body correlating with repression of the A-homoeolog. Colors indicate CG (green). CHG (blue), and CHH (red) methylation, and gene expression (black).

In the gene body, CG methlylation levels of most homoeologous genes were relatively equal (Fig 5A) and did not correlate with expression levels of homoeologous genes (Fig 5B). However, in fibers, we identified 539 pairs of A and D homoeologs that were differentially methylated at CHG and CHH sites in the gene body (Fig 5A). The methylation levels of CHG or CHH methylation in the gene body were significantly anti-correlated with expression levels of A and D homoeologs (Fig 5B and S5 Table). Among the genes that were hyper-methylated in the A relative to D homoeologs, nearly three times of D homoeologs were expressed at higher levels than the A homoeologs. Likewise, among the genes that were hyper-methylated in the D relative to A homoeologs, more than twice of A homoeologs were expressed at higher levels than the D homoeologs. For example, *A02G0163* was methylated more than *D02g0204*, and *D02G0204* was expressed more than *A02G0163* (Fig 5C). In the promoter regions, CG, CHG and CHH methylation levels of most homoeologous genes were relatively equal and did not show a significant correlation with gene expression (S5 Fig). Together, these data suggest a repressive role of CHG and CHH methylation in the gene body for the expression changes of homoeologous genes, which may contribute to fiber selection and improvement in the allotetraploid cotton.

## Discussion

We found little variation of CG and CHG methylation among cotton tissues, which is consistent with the data in maize, showing more variable CG and CHG methylation levels among genotypes than among tissues [40]. Although the overall level of CHH methylation is low compared with that of CG and CHG methylation in the genome, CHH methylation is associated with plant growth and development [41]. In *Arabidopsis* intraspecific hybrids, CHH methylation changes are associated with the parent-of-origin effect on circadian clock gene expression and biomass heterosis [41]. CHH methylation is mainly produced by 24-nt siRNAs through the RdDM pathway [1, 2]. In cotton, 24-nt siRNAs were enriched in gene-rich regions, which is similar to that in maize [28] and sugar beet [42] but different from that in *Arabidopsis* [43] and soybean [26]. Enrichment of siRNAs in euchromatic regions is inconsistent with the overall repeat abundance in the genome because the repeat amount is higher in soybean (~59%) [44] than in sugar beet (~42%) [42], but much lower than in maize (~85%) [45]. It is the TE property and location not the TE density that determines location of the siRNA abundance and CHH methylation distribution. These 24-nt siRNAs are derived from short TEs close to the genes, inducing CHH methylation flanking the genes, which is known as “CHH methylation island” in maize [28]. However, in spite of similar siRNA distribution patterns between maize and cotton, CHH methylation patterns are different. In maize, more siRNAs in gene-rich regions than TE-rich regions are associated with higher CHH methylation levels in gene-rich regions. In cotton, although gene rich regions also showed higher siRNA levels, CHH methylation level is lower in gene-rich regions than TE-rich regions (Fig 1C **)**. Furthermore, the percentage of methylcytosines in the CHH context is higher in cotton (~7.8% in leaves) than in maize (~5% in ears and shoots), which is in contrast to a higher TE density in maize than in cotton. These results indicate that in addition to CHH methylation that is produced by the RdDM pathway in cotton as in maize [10], cotton has another active pathway, which is absent in maize, to generate CHH methylation as shown in *Arabidopsis* [10].

This additional CHH hypermethylation in fibers is likely mediated by CMT2 and DDM1 [10]. The genes overlapping with FO CHH-hyper DMRs were not enriched in the differentially expressed genes, which is consistent with the finding that non-CG methylation generated by CMT2 does not regulate protein-coding genes [11]. The CHH hypermethylation in cotton fibers is probably promoted by concerted induction of *GhCMT2* and repression of *HISTONE H1*. In ovules, *GhCMT2* is also induced but *HISTONE H1* is not repressed. As a result, CHH methylation was not enhanced in the heterochromatin of ovules, suggesting that chromatin changes by histone H1 is required for promoting CHH hypermethylation in heterochromatin between the fiber and ovule. Fibers undergo endoreduplication in early stages and rapid cell elongation and cellulose synthesis in late stages, which may change chromatin structures in some regions [19, 20]. Indeed, more TEs are active in fibers than in ovules and leaves (Fig 4E), and more siRNAs were generated in fibers than other tissues [15]. The CHH hypermethylation induced in the fiber could function as a feedback mechanism to repress TEs. However, TEs are more expressed in fibers than in leaves, suggesting a paradox of the requirement of transcription for silencing, as previously noted [46]. Consistent with the requirement of DNA methylation for cotton fiber development, inhibiting DNA methylation by aza-dC not only reduces fiber cell initials but also slows down fiber cell elongation (Fig 4G, 4H and 4I). A confirmatory experiment is to generate the transgenic plants that suppress CHH methylation and directly test the CHH methylation effect on fiber development.

**Fig 6:**
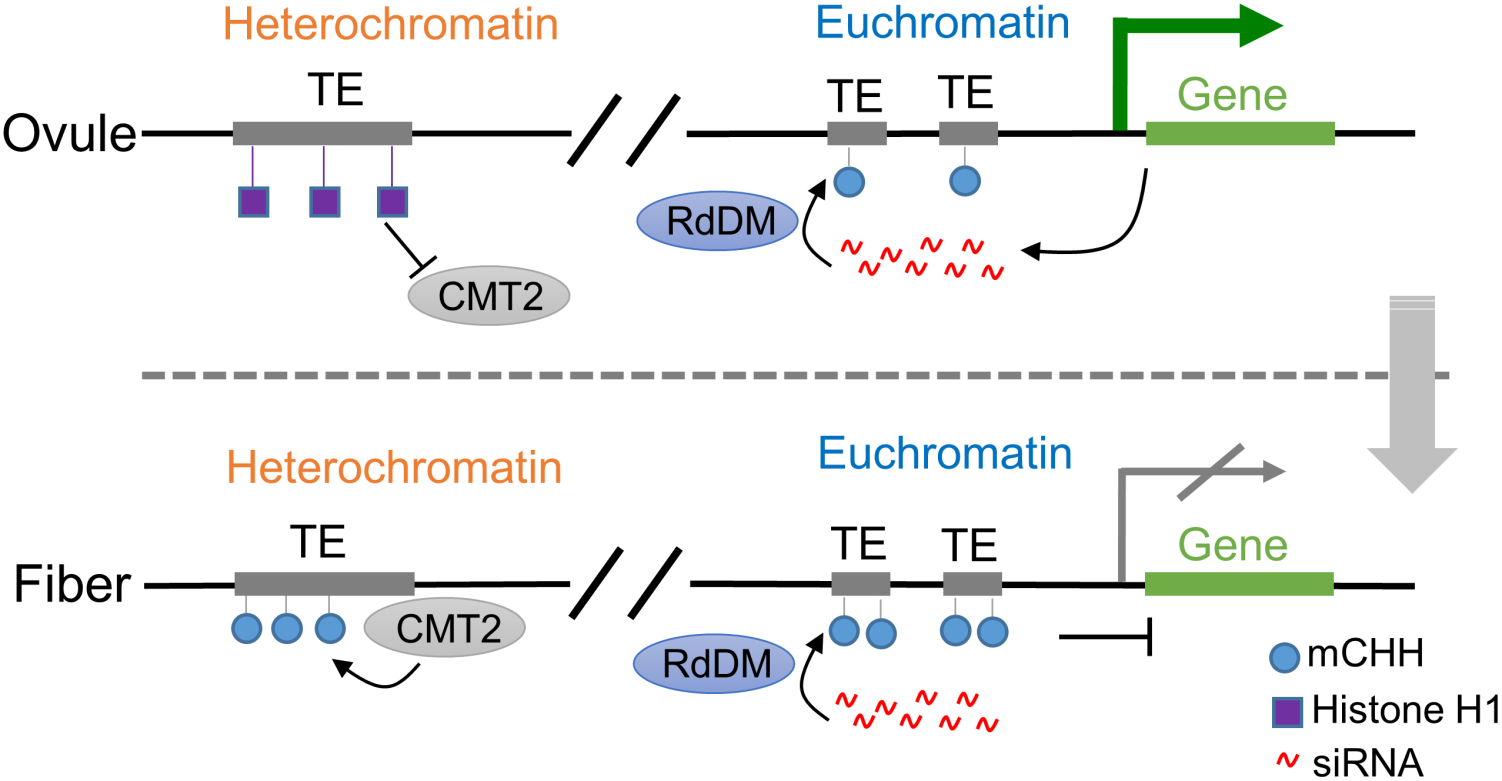
A model for CHH methylation and gene expression during ovule and fiber development. In ovules, transcription of genes in euchromatin induces activation of nearby TEs, leading to 24-nt siRNA production. These siRNAs promote CHH methylation of TEs through the RdDM pathway. However, occupation of histone H1 in heterochromatin impedes access of GhCMT2 to heterochromatin. During the fiber development, 24-nt siRNAs in euchromatin continue to induce additional CHH methylation of TEs near the genes, which eventually represses nearby genes. In heterochromatin where transcription of *HISTONE H1* is repressed, GhCMT2 could access TEs in heterochromatin to promote additional CHH methylation.

Notably, the highly expressed genes are correlated with higher CHH methylation levels in promoter regions close (1-kb or less) to the transcription start site (TSS), which is unexpected but has been documented in maize [28], soybean [26], rice [37], and now in cotton (Fig 3). One possibility is that TE activation enhances expression of nearby genes. Transcription initiates from TEs through Pol II or Pol IV (a homolog of Pol II) and then spreads to nearby genes [2, 47]. While most TEs are transcriptionally silenced in plant genomes, some TEs could activate nearby genes, as reported in *Arabidopsis* [46, 48] and rice [49]. If TE activation occurs prior to the transcription of nearby genes, TE expression should not be correlated with the distance between TEs and genes. However, more 24-nt siRNAs are present in the TEs closer to the genes (Fig 1G), suggesting another possibility that gene expression induces activation of nearby TEs. This may contribute to positive correlation between CHH methylation in the promoters and gene expression (Fig 6). The regions near the TSS are probably in open chromatin formation to allow active transcription of both genes by RNA polymerase II and short TEs by RNA polymerase IV [2, 47]. This leads to high abundance of small RNAs near the promoters and transcripts from corresponding genes, as observed in cotton, maize [28], and soybean [26]. The siRNAs can induce CHH methylation through the RdDM pathway. A recent study in rice showed that phosphate starvation induces DNA methylation of the TEs close to highly induced genes [37]. The methylation changes occur in the CHH context but are largely independent of the canonical RdDM pathway. Moreover, the methylation is increased after nearby genes are induced, suggesting that stress-induced gene expression promotes DNA methylation in the nearby TEs.

Consistent with this notion, ovule up-regulated genes are enriched in those overlapping with OL CHH-hyper DMRs in the flanking sequences. However, further CHH methylation of OL CHH-hyper DMRs in fibers represses nearby genes. This suggests that CHH methylation in promoters may act as a feedback mechanism to regulate these genes during ovule and fiber development (Fig 6). The spatiotemporal role of DNA methylation in expression changes of ovule- and fiber-related genes could also explain why overexpressing these genes may result in the unexpected outcome of fiber traits [24] because appropriate methylation patterns of the transgenes are not established. Together, the results suggest a functional role of CHH methylation during ovular and fiber development.

Finally, non-CG methylation in the gene body is associated with the expression bias of homoeologous genes in the allotetraploid cotton, providing the unique evidence for epigenetic regulation of nonadditive expression of homoeologous genes in polyploid species [50, 51]. Together, the spatiotemporal role of DNA methylation in developmental regulation and intergenomic interactions provides a conceptual advance, which could be translated into genomic improvement of polyploid plants, including most important crops that provide us with food (wheat), fiber (cotton), and oil (canola).

## Material and Methods

### Plant material

*Gossypium hirsutum* acc. TM-1 grew in the greenhouse at The University of Texas at Austin. Leaves and bolls (0 and 14 DPA) were harvested with three biological replications. Ovules and fibers were carefully dissected from cotton bolls at 14 DPA and immediately frozen in liquid nitrogen for RNA and DNA preparation.

### Small RNA-seq library construction

Total RNA was isolated from cotton leaves, ovules at 0 DPA and fibers at 14 DPA using Plant RNA Reagent (Life Technologies). By polyacrylamide gel electrophoresis, RNAs corresponding to ~15 to 30-nt in length from 20 μg total RNA were excised and eluted from the gel using 0.3 M NaCl. Small RNAs were precipitated using ethanol and dissolved in 6 μl RNase free water. Small RNA-seq libraries with three biological replicates were constructed using NEBNext^®^ Multiplex Small RNA Library Prep Set (NEB, Ipswich, Massachusetts) according to the manufacturer’s instructions. Small RNA-seq libraries were single-end sequenced for 50 cycles.

### mRNA-seq library construction

After DNase treatment, total RNA (~5 μg) was subjected to construct strand-specific mRNA-seq libraries with two biological replications using NEBNext^®^ Ultra^™^ Directional RNA Library Prep Kit (NEB, Ipswich, Massachusetts) according to the manufacturer’s instructions. mRNA-seq libraries were single-end sequenced for 150 cycles.

### MethylC-seq library construction

Genomic DNA was isolated from cotton leaves, ovules at 0 DPA and 14 DPA, and fibers at 14 DPA using CTAB method [52]. Total genomic DNA (~5 μg) was fragmented to 100–1000 bp using Bioruptor (Diagenode, Denville, New Jersey). End repair (NEBNext^®^ End Repair Module) was performed on the DNA fragment by adding an 'A' base to the 3'end (NEBNext^®^ dA-Tailing Module), and the resulting DNA fragment was ligated to the methylated DNA adapter (NEXTflex^™^ DNA Barcodes, Bioo Scientific, Austin, Texas). The adapter-ligated DNA of 200–400 bp was purified using AMPure beads (Beckman Coulter, Brea, California), followed by sodium bisulfite conversion using MethylCode^™^ Bisulfite Conversion Kit (Life Technologies, Foster City, California). The bisulfite-converted DNA was amplified by 12 cycles of PCR using LongAmp^®^ *Taq* DNA Polymerase (NEB, Ipswich, Massachusetts) and purified using AMPure beads (Beckman Coulter, Brea, California). The Paired-End sequencing of the MethylC-seq libraries was performed for 101 cycles.

### qRT-PCR

After DNase treatment, total RNA (2 μg) was used to produce first-strand cDNA with the Omniscript RT Kit (Qiagen, Valencia, California). The cDNA was used as the template for qRT–PCR using FastStart Universal SYBR Green Master (Roche, Indianapolis, Indiana). The reaction was run on the LightCycler^®^ 96 System (Roche, Pleasanton, California). The relative expression level was quantified using internal control cotton *HISTONE H3* [53].

### Small RNA-seq data analysis

After adapter clipping, small RNA-seq reads (18–30 nt) were mapped to the TM-1 genome sequence [34] using Bowtie2 settings (-k 100—score-min L,0,0), allowing no mismatches. Multi-mapped small RNA-seq reads were evenly weighted and assigned to all locations as previously described method [54].

### mRNA-seq data analysis

mRNA-seq reads were mapped to the TM-1 genome sequence using Tophat settings (—library-type fr-firststrand —b2-score-min L,0,-0.2) [34, 55]. Uniquely mapped reads were extracted and analyzed by Cufflinks to determine gene and TE abundance using annotated genes and TEs [56]. The differentially expressed genes (DEGs) were identified using both the fold-change (>2-fold) and ANOVA tests (*P* < 0.01).

### Identification of methylated cytosines

We applied Bismarck software to align reads to the TM-1 genome sequence with default parameters (—score_min L,0,-0.2 -X 1000—no-mixed—no-discordant) [34, 57]. In brief, the first 75 bases of unmapped reads were extracted and realigned to the genome. Only reads mapped to the unique sites were retained for further analysis. Reads mapped to the same site were collapsed into a single consensus read to reduce clonal bias. For each cytosine, the binomial distribution was used to identify whether this cytosine was methylated. The probability *p* in binomial distribution B (n, *p*) was referred to bisulfite conversion failure rate. The number of trials (n) in the binomial distribution was the read depth. Only the cytosines covered by at least three reads in all compared tissues were considered for further analysis. Cytosines with P-values below 1e^–5^ were identified as methylcytosines.

### Differentially methylated regions

Differentially methylated regions (DMRs) for CHH methylation were identified using 100-bp sliding-windows. Mean methylation level was calculated for each window [5]. Windows containing at least eight cytosines in the CHH context covered by at least three reads were selected for identifying DMRs. ANOVA test using 2 biological replications (*P* < 0.05) and difference of methylation levels (cut-off value > 0.1 between two compared samples) were used to determine CHH DMRs between two compared tissues. The cut-off value was set at 0.05 for the comparison between ovule and fiber methylation levels in OL CHH-hyper DMRs.

The fold enrichment of DEGs in DMR-overlapping genes was calculated as (#DEGs_DMR-overlapping_ /#DEGs_total_) / (#Genes _DMR-overlapping_ /#Genes_total_), and *P*-values were generated using the hypergeometric test.

### Homoeologous genes

Protein sequences of A homoeologs were aligned to protein sequences of D homoeologs using blastp with an E-value less than 1e^-10^. Alignment information was used by MCScanx to identify homoeologous genes [58] (Score > 2000 and E-value < 1e^-10^).

### Z-score for enrichment of DMRs flanking genes

We first randomly extracted 1000 of 1-kb regions in the genome for 500 times. Percentage of DMR-overlapping regions in 1000 random regions was calculated at each time. Z-scores were calculated as (*x*-*μ*)/*σ* where x was percentage of DMR-flanking genes in all genes, *μ* and *σ* were mean and standard deviation of percentages of DMR-overlapping regions in random loci.

### Cotton ovule in vitro culture and 5-aza-dC treatment

Ovules were removed from flower buds at -3 or 0 DPA. The ovules were sterilized with 75% alcohol, washed with autoclaved water, and cultured in Beasley-Ting (BT) liquid media (20 ml) containing 5 μM indole-3-acetic acid (IAA) and 0.5 μM gibberellic acid3 (GA3) at 30°C for 4–14 days in dark [17, 59]. Treatment of 5-aza-2’-deoxycytidine (aza-dC, 10 mg/L) was applied in the BT liquid media.

### Accession numbers

The data reported in this paper have been deposited in the Gene Expression Omnibus (GEO) database, www.ncbi.nlm.nih.gov/geo (accession no. GSE61774).

## Supporting Information

S1 Fig Genome wide distribution of DNA methylation in leaf, ovule and fiber. (A) Pearson correlation of biological replications in differentially methylated cytosines (DmCs). (B) Percentage of methylated cytosine (mC) in A-genome and D-genome in different tissues. A and D indicate A and D subgenomes in allotetraploid cotton, respectively. (C) Circle plots of DNA methylation, gene density, TE density and siRNA abundance of cotton ovules and leaves in 13 homoeologous chromosomes in the A subgenome (left) and 13 homoeologous chromosomes in the D subgenome (right). (D) Correlation of CHH methylation difference with TE density between fiber and ovule (left) and between ovule and leaf (right).
(PDF)

S2 Fig Distribution of CG and CHG methylation in gene and TE. (A) Distribution of CG and CHG methylation in gene region. (B) Distribution of CG and CHG methylation in TE region.
(PDF)

S3 Fig Bisulfite sequencing results of randomly selected DMRs. (PDF)

S4 Fig Correlation of DNA methylation and gene expression. (A) DNA methylation in all contexts throughout genes divided into four quartiles based on gene expression in leaf. (B) DNA methylation in all contexts throughout genes divided into four quartiles based on gene expression in fiber.
(PDF)

S5 Fig DNA methylation differences in promoters of homoeologous genes. (A) Pairwise plots of CG (left), CHG (middle), and CHH (right) methylation levels (Y-axis, D homoeologs; X-axis, A homoeologs) in promoter regions (2-kb) of homoeologous genes. Blue and red dots indicate the expression level of an A homoeolog that is lower and higher than that of the corresponding D homoeolog, respectively. Spearman correlation coefficient (r) is shown. (B) Number of homoeologous genes that were differentially methylated in CG (left), CHG (middle), and CHH (right) sites in promoter regions and showed expression differences (A<D, blue; A>D, red). The levels of CG (>0.4), CHG (>0.4), and CHH (>0.1) methylation difference and fold-changes of expression (>2-fold) between A and D homoeologs were used as cut-off values.
(PDF)

S1 Table Summary of high-throughput sequencing reads. (XLSX)

S2 Table OL and FO CHH-hyper DMRs. (XLSX)

S3 Table OL CHH-hyper DMRs overlapping genes. (XLSX)

S4 Table FO CHH-hyper DMRs overlapping genes. (XLSX)

S5 Table Homoeologous genes with differentially methylated gene body. (XLSX)
