# Supplementary material for: Dynamic Roles for Small RNAs and DNA Methylation during Ovule and Fiber Development in Allotetraploid Cotton
Source: PLoS Genet. 2015 Dec 28;11(12):e1005724. doi: 10.1371/journal.pgen.1005724 (PMC4692501; doi:10.1371/journal.pgen.1005724)
Supplement: S1 Fig — (A) Pearson correlation of biological replications in differentially methylated cytosines (DmCs). (B) Percentage of methylated cytosine (mC) in A-genome and D-genome in different tissues. A and D indicate A and D subgenomes in allotetraploid cotton, respectively. (C) Circle plots of DNA methylation, gene density, TE density and siRNA abundance of cotton ovules and leaves in 13 homoeologous chromosomes in the A subgenome (left) and 13 homoeologous chromosomes in the D subgenome (right). (D) Correlation of CHH methylation difference with TE density between fiber and ovule (left) and between ovule and leaf (right). (PDF) [file pgen.1005724.s001.pdf]

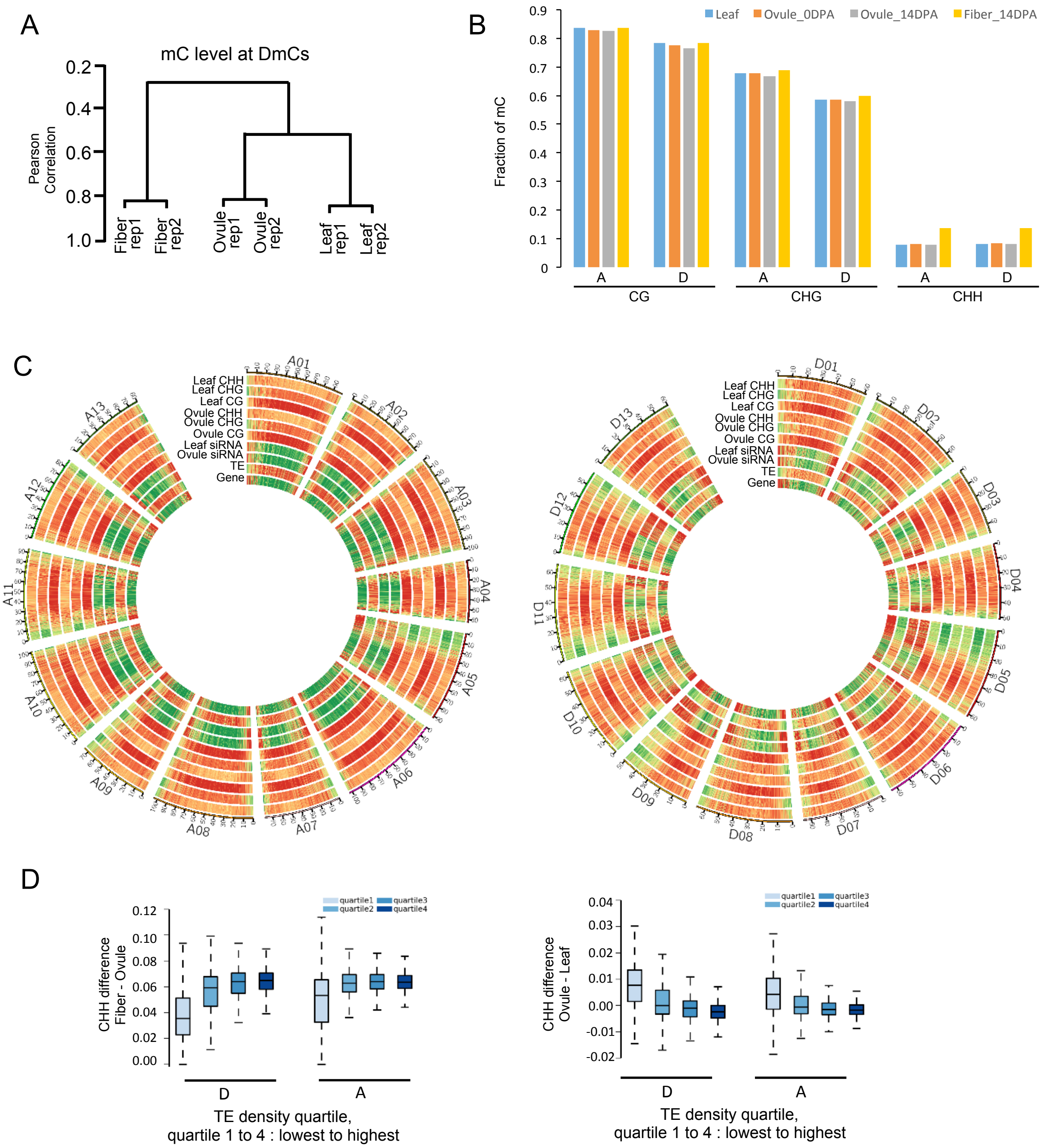

S1 Fig. Genome wide distribution of DNA methylation in leaf, ovule and fiber. (A) Pearson correlation of biological replications in differentially methylated cytosines (DmCs). (B) Percentage of methylated cytosine (mC) in A-genome and D-genome in different tissues. A and D indicate A and D subgenomes in allotetraploid cotton, respectively. (C) Circle plots of DNA methylation, gene density, TE density and siRNA abundance of cotton ovules and leaves in 13 homoeologous chromosomes in the A subgenome (left) and 13 homoeologous chromosomes in the D subgenome (right). (D) Correlation of CHH methylation difference with TE density between fiber and ovule (left) and between ovule and leaf (right).
