# Supplementary material for: Dynamic Roles for Small RNAs and DNA Methylation during Ovule and Fiber Development in Allotetraploid Cotton
Source: PLoS Genet. 2015 Dec 28;11(12):e1005724. doi: 10.1371/journal.pgen.1005724 (PMC4692501; doi:10.1371/journal.pgen.1005724)
Supplement: S2 Fig — (A) Distribution of CG and CHG methylation in gene region. (B) Distribution of CG and CHG methylation in TE region. (PDF) [file pgen.1005724.s002.pdf]

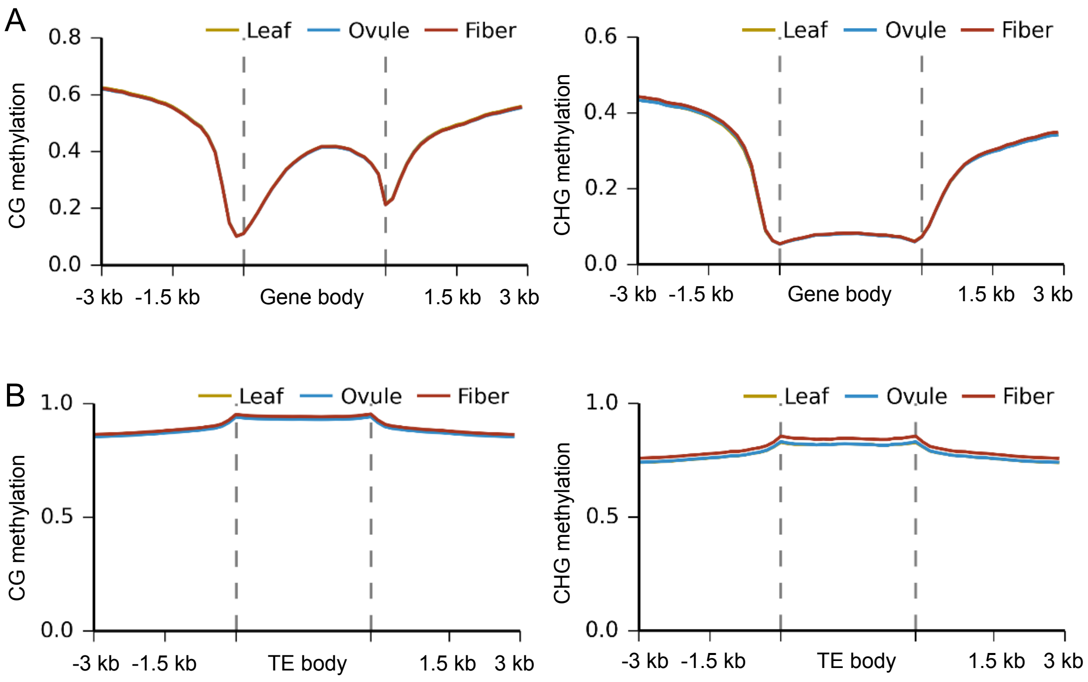

S2 Fig. Distribution of CG and CHG methylation in gene and TE. (A) Distribution of CG and CHG methylation in gene region. (B) Distribution of CG and CHG methylation in TE region.
