# Supplementary figures and images for: Dynamic Roles for Small RNAs and DNA Methylation during Ovule and Fiber Development in Allotetraploid Cotton
Source: PLoS Genet. 2015 Dec 28;11(12):e1005724. doi: 10.1371/journal.pgen.1005724 (PMC4692501; doi:10.1371/journal.pgen.1005724)

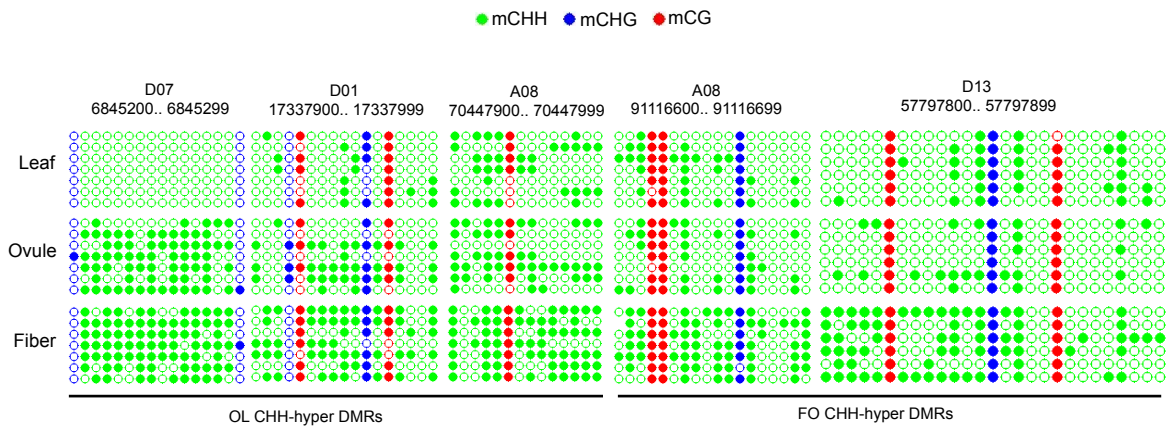

**S3 Fig. Bisulfite sequencing results of randomly selected DMRs.**

Supplement: S3 Fig — (PDF) [file pgen.1005724.s003.pdf]
