# Supplementary material for: Dynamic Roles for Small RNAs and DNA Methylation during Ovule and Fiber Development in Allotetraploid Cotton
Source: PLoS Genet. 2015 Dec 28;11(12):e1005724. doi: 10.1371/journal.pgen.1005724 (PMC4692501; doi:10.1371/journal.pgen.1005724)
Supplement: S4 Fig — (A) DNA methylation in all contexts throughout genes divided into four quartiles based on gene expression in leaf. (B) DNA methylation in all contexts throughout genes divided into four quartiles based on gene expression in fiber. (PDF) [file pgen.1005724.s004.pdf]

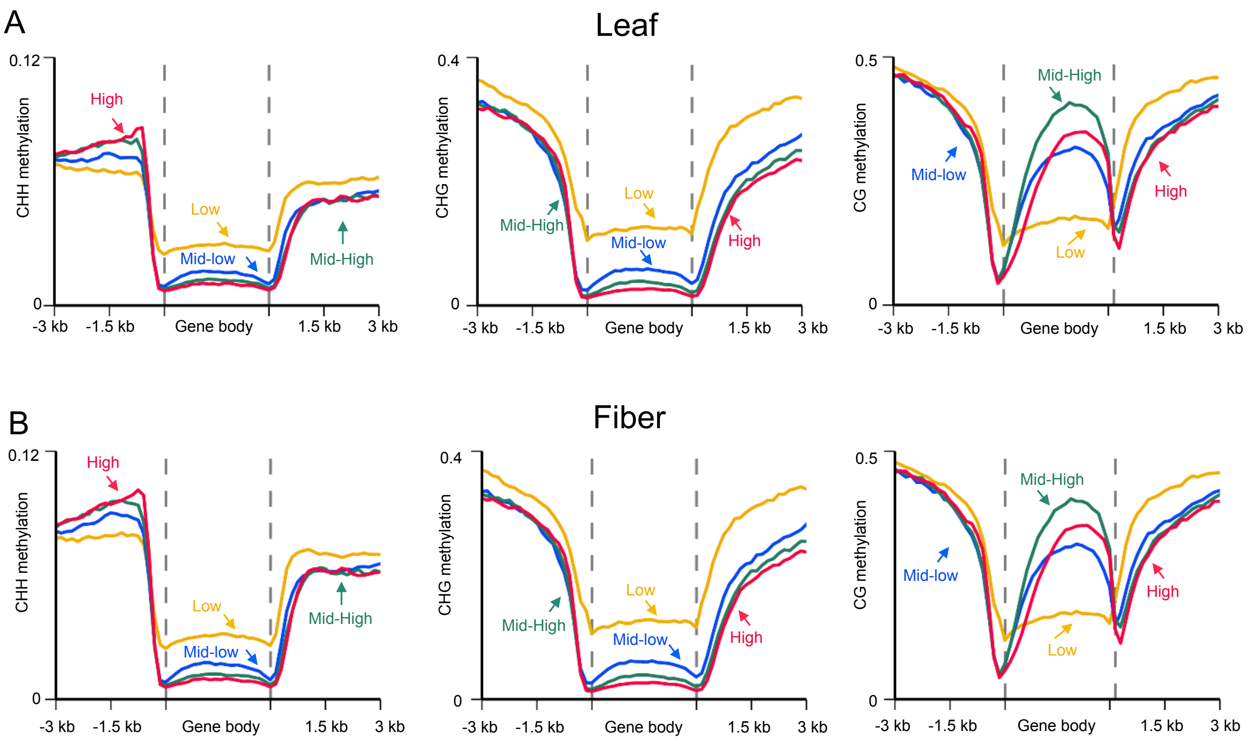

S4 Fig. Correlation of DNA methylation and gene expression. (A) DNA methylation in all contexts throughout genes divided into four quartiles based on gene expression in leaf. (B) DNA methylation in all contexts throughout genes divided into four quartiles based on gene expression in fiber.
