# Supplementary material for: Dynamic Roles for Small RNAs and DNA Methylation during Ovule and Fiber Development in Allotetraploid Cotton
Source: PLoS Genet. 2015 Dec 28;11(12):e1005724. doi: 10.1371/journal.pgen.1005724 (PMC4692501; doi:10.1371/journal.pgen.1005724)
Supplement: S5 Fig — (A) Pairwise plots of CG (left), CHG (middle), and CHH (right) methylation levels (Y-axis, D homoeologs; X-axis, A homoeologs) in promoter regions (2-kb) of homoeologous genes. Blue and red dots indicate the expression level of an A homoeolog that is lower and higher than that of the corresponding D homoeolog, respectively. Spearman correlation coefficient (r) is shown. (B) Number of homoeologous genes that were differentially methylated in CG (left), CHG (middle), and CHH (right) sites in promoter regions and showed expression differences (A<D, blue; A>D, red). The levels of CG (>0.4), CHG (>0.4), and CHH (>0.1) methylation difference and fold-changes of expression (>2-fold) between A and D homoeologs were used as cut-off values. (PDF) [file pgen.1005724.s005.pdf]

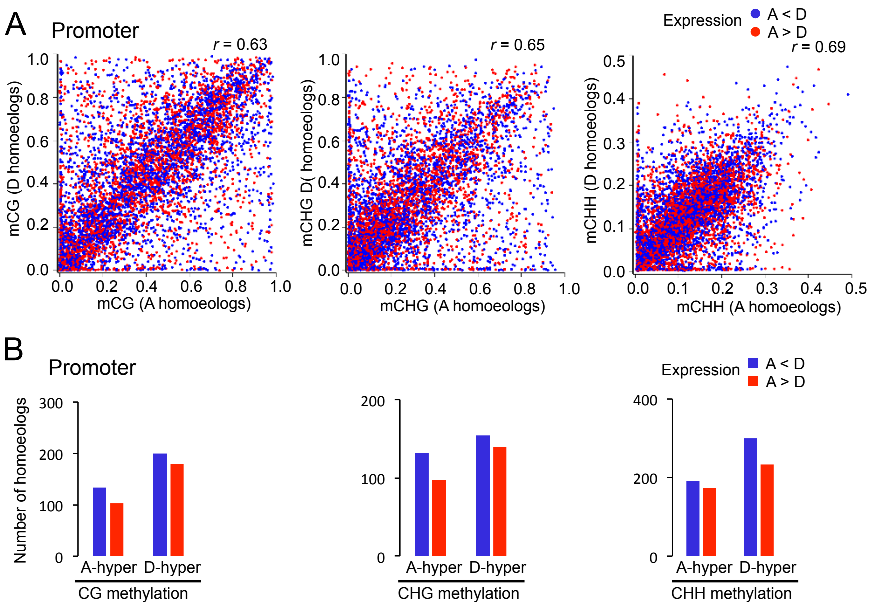

S5 Fig. DNA methylation differences in promoters of homoeologous genes. (A) Pairwise plots of CG (left), CHG (middle), and CHH (right) methylation levels (Y-axis, D homoeologs; X-axis, A homoeologs) in promoter regions (2-kb) of homoeologous genes. Blue and red dots indicate the expression level of an A homoeolog that is lower and higher than that of the corresponding D homoeolog, respectively. Spearman correlation coefficient (r) is shown. (B) Number of homoeologous genes that were differentially methylated in CG (left), CHG (middle), and CHH (right) sites in promoter regions and showed expression differences (A<D, blue; A>D, red). The levels of CG (>0.4), CHG (>0.4), and CHH (>0.1) methylation difference and fold-changes of expression (>2-fold) between A and D homoeologs were used as cut-off values.
